# Combined effects of warming and hypoxia on early life stage Chinook salmon physiology and development

**DOI:** 10.1093/conphys/coy078

**Published:** 2019-02-18

**Authors:** Annelise M Del Rio, Brittany E Davis, Nann A Fangue, Anne E Todgham

**Affiliations:** 1 Department of Animal Science, University of California Davis, Davis, CA, USA; 2 Department of Wildlife, Fish, and Conservation Biology, University of California Davis, Davis, CA, USA; 3 California Department of Water Resources, Division of Environmental Services, PO Box 942836, Sacramento, CA, USA

**Keywords:** Chinook salmon, climate change, developmental physiology, hypoxia, temperature

## Abstract

Early life stages of salmonids are particularly vulnerable to warming and hypoxia, which are common stressors in hyporheic, gravel bed, rearing habitat (i.e. a ‘redd’). With the progression of global climate change, high temperatures and hypoxia may co-occur more frequently within redds, particularly for salmonid species at their southern range limit. Warming and hypoxia have competing effects on energy supply and demand, which can be detrimental to energy-limited early life stages. We examined how elevated temperature and hypoxia as individual and combined stressors affected the survival, physiological performance, growth, and development of Chinook salmon (*Oncorhynchus tshawytscha*). We reared late fall-run Chinook salmon from fertilization to the fry stage in a fully factorial design of two temperatures [10°C (ambient) and 14°C (warm)] and two oxygen levels [normoxia (100% air saturation, 10 mg O_2_/l) and hypoxia (50% saturation, 5.5 mg O_2_/l)]. Rearing in hypoxia significantly reduced hatching success, especially in combination with warming. Both warming and hypoxia improved acute thermal tolerance. While rearing in hypoxia improved tolerance to acute hypoxia stress, warming reduced hypoxia tolerance. Hypoxia-reared fish were smaller at hatch, but were able to reach similar sizes to the normoxia-reared fish by the fry stage. High temperature and normoxia resulted in the fastest rate of development while low temperature and hypoxia resulted in the slowest rate of development. Despite improved physiological tolerance to acute heat and hypoxia stress, hypoxia-reared embryos had reduced survival and growth, which could have larger population-level effects. These results suggest that both warming and hypoxia are important factors to address in conservation strategies for Chinook salmon.

## Teaser

Salmonids are particularly susceptible to warming and hypoxia during development in redds. We reared Chinook salmon embryos and alevins under chronic warming and hypoxia to evaluate the effects of each stressor individually and their interaction. Warming and hypoxia affected survival, physiological performance and development with management implications for salmon conservation.

## Introduction

Increasing water temperatures resulting from climate change are predicted to be problematic for numerous species, particularly for fishes such as Pacific salmonids, which require cool, flowing, highly oxygenated water (Moyle, 2002). The Central Valley watershed of California supports the southernmost populations of Chinook salmon (*Oncorhynchus tshawytscha)*, and is projected to see large, consistent temperature increases nearing 5°C this century (Hayhoe *et al.*, 2004; Dettinger, 2005). In addition to warming, hypoxia (low dissolved oxygen [DO] in the environment) is rapidly becoming more prevalent globally because of climate change and anthropogenic influences, such as eutrophication from agriculture and sewage runoff (Diaz, 2001; Breitburg *et al.*, 2018). Warming and hypoxia are likely to co-occur, as oxygen is less soluble in warmer water (Keeling *et al.*, 2010; Helm *et al.*, 2011). In California, the effects of climate change have been exacerbated by prolonged drought, as warming and low water flows increase water temperatures and thus the potential for hypoxia to occur (Hanak *et al.*, 2015). While the effects of each stressor on animal physiology have been studied in depth individually, there is a greater need to study the interaction between the two stressors in environmentally relevant scenarios (Crain *et al.*, 2008; Todgham and Stillman, 2013; Gunderson *et al.*, 2016).

Both warming and hypoxia are common stressors within the microhabitat of salmon redds, the gravel nests where embryos and larvae develop within the streambed. Temperature and DO within redds are influenced by numerous abiotic and biotic factors including intragravel flow velocity, sedimentation, gravel size, groundwater upwelling and oxygen consumption by developing embryos or other organic matter present (Acornley, 1999; Greig *et al.*, 2007a; Sear *et al.*, 2014). Hypoxia within redds has been correlated with detrimental effects on survival and growth of developing salmonids in natural streams (Rubin and Glimsäter, 1996; Youngson *et al.*, 2004; Greig *et al.*, 2007b). In California, as with many river systems, the rearing conditions for egg and fry development are determined by water releases from dams upstream. These water releases further dictate key abiotic water parameters downstream including temperature and DO. The combination of warming and low DO as a result of low water flows is thought to have reduced the thermal tolerance, and thus survival, of Chinook salmon embryos in the Sacramento River (Martin *et al.*, 2017).

From a physiological perspective, warming and hypoxia are likely to interact through contrasting effects on energy metabolism. Temperature is a controlling factor that determines metabolic rates in ectotherms, whereas oxygen is a limiting factor that restricts metabolic rate (Fry, 1971). Therefore, as warming increases metabolism, hypoxia limits the oxygen supply available to support increased metabolic demand (McBryan *et al.*, 2013). The concept of oxygen and capacity limitation of thermal tolerance (OCLTT) hypothesizes that the mismatch in oxygen supply and demand can reduce thermal tolerance and affect the physiology and ecology of many species (Pörtner, 2001). The OCLTT hypothesis predicts temperature and oxygen will interact negatively to influence stress tolerance such that exposure to high temperature is expected to reduce hypoxia tolerance and hypoxia is expected to reduce thermal tolerance (McBryan *et al.*, 2013).

Early life stages of Chinook salmon are particularly sensitive to both warming and hypoxia, as embryos and alevins are the least thermally tolerant life stages and have little to no mobility to avoid suboptimal habitat conditions (McCullough, 1999; Myrick and Cech, 2004). Embryos of oviparous fish such as salmonids have stronger energy constraints than older organisms because they possess a finite amount of energy in the form of yolk to support their development (Rombough, 2006). Under optimal conditions during development, the majority of energy is allocated towards growth. When energy supply or demand is altered, as with warming or hypoxia, there is increased competition for energy between coping with stress and continued growth and development (Sokolova, 2013). With a limited ability to increase aerobic metabolic rate above routine levels, compensatory energy partitioning may detract energy from processes necessary for development (Rombough, 2011). Therefore, the metabolic interactions between warming and hypoxia may be especially detrimental during early development.

Developing salmon are known to be sensitive to warming and hypoxia individually but are likely to experience both stressors simultaneously in their rearing environment, especially as climate change progresses and local anthropogenic impacts (e.g. drought) persist. In this study, we assessed the effects of chronic warming and hypoxia, on developing late fall-run Chinook salmon, as individual and combined stressors. We reared salmon from fertilization through the fry stage in a fully factorial design of two temperatures (10 and 14°C) and two oxygen levels (100% and 50% air saturation). Throughout development we measured hatching success, growth and developmental rate as well as tolerance to acute thermal and hypoxic stress to examine the lethal and sublethal responses to rearing in each treatment. We predicted that there would be detrimental effects of warming and hypoxia as individual stressors that would be amplified through synergistic interactions in the multiple stressor treatment due to competing effects on balancing energy supply and demand. Examining the effects of two key stressors across salmon development will further our understanding of the capacity of early life stage salmonids to cope with multiple stressors in their natural environment. Our findings can also inform water management strategies to promote egg to fry survival in highly managed, complex systems such as the Sacramento River.

## Materials and methods

### Fish acquisition and care

Freshly fertilized late fall-run Chinook salmon embryos were obtained from four separate breeding pairs spawned at the Coleman National Fish Hatchery (US Fish and Wildlife Service, Anderson, CA, USA). Embryos were transported to the University of California Davis Center for Aquatic Biology and Aquaculture in January 2017. Embryos were immediately transferred to their rearing treatments in one of four replicate 19 l square culture buckets. Embryos were held in floating mesh baskets affixed with plastic dividers creating individual wells to keep embryos separated in an even layer. Embryos from all four families were evenly distributed across each replicate bucket. Once alevins could sustain swimming, the baskets were removed from the culture buckets. Since early developmental stages rely on endogenous yolk reserves (Kamler, 2008), fish were not fed during the experiment. The experiment ended when fish reached the fry stage and nearly all of the yolk sac was absorbed. All fish care and protocols were reviewed and approved by the UC Davis Institutional Animal Care and Use Committee (protocol no. 19 593).

### Experimental design

To assess the effects of elevated temperature and decreased oxygen as individual and combined stressors, we reared developing Chinook salmon from fertilization to the fry stage in four treatments in a fully factorial design of two temperatures [10°C (ambient) and 14°C (warm)] and two oxygen (O_2_) saturation levels [normoxia (100% air saturation, 10 mg O_2_/l) and hypoxia (50% saturation, 5.5 mg O_2_/l)]. Ambient temperature of 10°C was chosen as this is within the average range of temperatures in the Sacramento River when late fall-run salmon embryos are present (Bureau of Reclamation (2018), Central Valley Operations, Sacramento River Temperature Report). The warm temperature of 14°C was chosen to represent a 4°C increase of water temperatures projected with climate change and is a potentially stressful, but not lethal, temperature because embryo mortality increases around 16°C in California Chinook salmon (Myrick and Cech, 2004; Williams, 2006). Dissolved oxygen within natural redds can fluctuate widely between 2–11 mg O_2_/l (Coble, 1961; Peterson and Quinn, 1996). Normoxia was maintained at 100% saturation to represent optimal habitat conditions and 50% was chosen as a moderate level of hypoxia that is potentially stressful, but not lethal (Silver *et al.*, 1963). Two different temperature treatments were maintained by placing culture and reservoir buckets in four large water bath tanks (1.2 m in diameter) containing flow through water at the corresponding treatment temperature. Each water bath (at 10°C or 14°C) held two culture buckets from the normoxia and hypoxia treatments, with two replicate water bath tanks for each temperature (*n* = 4 culture buckets per temperature and DO treatment combination). Oxygen saturation was manipulated using mass flow controller valves (Sierra Instruments, Monterey, CA, USA) to mix N_2_ gas and air to maintain low DO in hypoxic treatments or air alone for normoxic treatments. The gas mixture was bubbled into reservoir buckets using venturi injectors (one reservoir bucket for each temperature × DO treatment). Equilibrated treatment water from each reservoir was then dripped into the culture buckets holding salmon at 16 l/h to ensure high turnover. Gas mixtures were also bubbled directly into culture buckets through air stones for further mixing and adjustment of DO levels within each individual bucket. Temperature and DO were measured in each culture bucket, reservoir bucket, and water bath tank daily using a handheld meter (OxyGuard Handy Polaris 2, OxyGuard International, Farum, Denmark), summarized in Table 1.

**Table 1:** Water temperature (°C) and dissolved oxygen (DO, mg/l and % saturation) in each treatment for the duration of the experiment. Water temperature was measured daily in each water bath tank and is reported as the average between the duplicate tanks for each temperature treatment (±SD). Dissolved oxygen was measured daily in each culture bucket and is reported as the average of the four replicate culture buckets per treatment (±SD).

Treatment	Temperature (°C)	DO (mg/l)	DO % saturation
14°C Normoxia	14.1 ± 0.7	10.1 ± 0.5	98.2 ± 4.2
14°C Hypoxia	14.1 ± 0.7	5.9 ± 3.1	55.3 ± 7.2
10°C Normoxia	10.6 ± 0.9	10.8 ± 0.4	97.5 ± 3.2
10°C Hypoxia	10.6 ± 0.9	5.5 ± 0.8	49.9 ± 7.6

Physiological testing occurred four times during the study period for each treatment. A stage-based sampling design was chosen to account for differences in developmental rate caused by the varying temperatures and oxygen saturation levels between treatments. Sampling took place when 50% or more of embryos in a treatment reached (i) eyed stage, when dark pigmented eyes were clearly visible, (ii) silver eyed stage, when silver pigment in eyes was visible, (iii) alevin stage, 1 day after hatching and lastly (iv) fry stage, when the yolk sac was almost completely absorbed. Development of salmon was monitored daily with visual inspections of each culture bucket. Stage was assessed at the treatment level because families were equally distributed among replicates, contributing to minimal variation in developmental timing between replicates. Hatching success was calculated as the ratio between the number of alevins 1-day post-hatch and the initial number of embryos per treatment. Upper thermal tolerance was assessed at each stage (eyed, silver-eyed, alevin and fry) as critical thermal maximum (CTMax), and hypoxia tolerance (time to loss of equilibrium) was tested for fry only. At the alevin and fry stages total length and mass were recorded.

### Determination of upper thermal tolerance

Acute upper thermal tolerance was measured using critical thermal maximum (CTMax) methodology (Beitinger *et al.*, 2000; Fangue *et al.*, 2006). All CTMax trials were conducted in normoxic water (100% air saturation). The endpoint used to indicate CTMax differed between embryos and post-hatch stages due to the inability of embryos to exhibit loss of equilibrium, a common endpoint for fishes after hatch (Zebral *et al.*, 2018).

#### Embryos

Critical thermal maximum for embryos at the eyed and silver eyed stages was defined as the temperature at which the heart stopped beating, similar to Angilletta *et al.* (2013). CTMax was determined in four embryos from each of four replicates per treatment (16 embryos total per treatment). Embryos were placed in individual wells of a divided plastic dish with water at their corresponding rearing temperature. The plastic dish was held in a well of an aluminum block and treatment water was circulated through the aluminum block to maintain treatment temperature. Embryos were given 1 h in the dishes before CTMax trials began (Becker and Genoway, 1979). Circulating water was then heated using a submersible heater and YSI Thermistemp Temperature Controller (YSI Incorporated, Yellow Springs, OH, USA) such that the water temperature in the dish increased at a rate of 0.3°C/min. Water was aerated using a pipette to ensure full oxygenation and circulation. Embryos were continuously monitored under a dissecting microscope and CTMax was recorded as the temperature when the heart was observed to stop beating for more than 30 s.

#### Alevins and fry

For alevins and fry, CTMax was determined for four fish per replicate per treatment (16 fish total per treatment). The apparatus consisted of a 37 l aquarium containing a water heater connected to a YSI Thermistemp Temperature Controller (YSI Incorporated), a submersible pump for circulation, and eight glass chambers suspended in the aquaria. Individual fish were placed in the jars for 1 h prior to the start of each trial with water at the corresponding rearing temperature. Eight fish were run at a time and jars were each continuously aerated throughout the CTMax protocol to ensure full oxygenation. After the 1 h acclimation, the heater was turned on and the water temperature increased at a rate of 0.3°C/min. Fish were closely monitored until they reached loss of equilibrium (LOE), defined as the point at which a fish could no longer swim upright or respond to a gentle physical stimulus. Temperature at LOE was recorded with a calibrated immersion thermometer (0.1°C precision, Fisher Scientific), after which individuals were immediately transferred to a fully oxygenated recovery tank with water at their rearing temperature. Temperature at LOE was included in the final dataset if the individual survived a 24 h recovery period. This protocol ensures that CTMax is not exceeded and therefore overestimated. One alevin in the 10°C normoxia treatment died during recovery, whereas 16 of the fry in the 14°C normoxia treatment that underwent the CTMax trial died during recovery. As a result, an additional 8 fish in this treatment were tested the same day to ensure an adequate sample size (*n* = 8 for 14°C normoxia fry).

### Fry hypoxia tolerance

Acute hypoxia tolerance of salmon fry was measured using time to loss of equilibrium methodology (Anttila *et al.*, 2015, McBryan *et al.*, 2016). Time to LOE was determined for four fish per replicate per treatment (16 fish per treatment). Hypoxia tolerance trials were conducted in a 37 l aquarium held in a temperature-controlled water bath. The aquarium contained eight floating plastic beakers with mesh sides for individual fry and a submersible pump for water circulation. The water surface within each beaker was covered with bubble wrap to prevent surface respiration during trials. The water surface surrounding the beakers was also covered with bubble wrap to prevent diffusion of oxygen into the water during trials. DO was monitored throughout the trial using two oxygen dipping probes (PreSens Precision Sensing, Regensburg, Germany). Individual fish were placed in each beaker 30 min prior to the start of the trial to recover from handling. Fish were tested in water at the same temperature and DO level as their rearing treatment. In each trial DO of the water was reduced at a rate of 1.5–2%/min from initial oxygen levels (i.e. 100% and 50%) by bubbling in N_2_ gas until 8% air saturation was reached (0.9 mg O_2_/l at 10°C and 0.8 mg O_2_/l at 14°C). Oxygen was then held at 8% by manually adjusting the flow of N_2_. This final oxygen concentration was chosen based on pilot studies where all fish could maintain equilibrium indefinitely at 10% and there was little variation in the rapid time to LOE at 6%. Time to LOE was defined as the time (min) after DO saturation reached 8% until the fish could no longer swim upright or respond to a gentle physical stimulus. Upon achieving LOE fish were immediately transferred to fully oxygenated recovery chambers at respective rearing temperatures. Each trial was conducted with a maximum trial time of 2 h. Fish that maintained equilibrium when the 2 h trial ended were assigned a time to LOE of 120 min and transferred to recovery. Time to LOE for fish that survived a 24 h recovery period was included in the final dataset. A total of five fish did not survive recovery, three from the 14°C normoxia treatment, and one each in the 14°C hypoxia and 10°C normoxia treatments.

### Body condition factor

Fish at the alevin and fry stages (*n* = 5 per replicate tank, *n* = 20 total per treatment) were euthanized in tricaine methanesulfonate (MS-222, Western Chemical, Ferndale, WA, USA), weighed, and measured for total length. Alevin mass measurements included the yolk sac. Body condition was used to compare overall size differences between treatment. Fulton’s condition factor (K) was calculated as:

$$K\;=\;100\;x\;\frac{W}{L^{3}}$$

where *W* is the wet mass in grams and *L* is the total length of the fish in cm.

### Statistical analyses

Statistical analyses were performed using R Studio (v3.3.0, R Development Core Team, 2013). Datasets were visually inspected for assumptions of normality and homogeneity of variances using Q-Q plots and residuals vs. fitted values. All data were normally distributed and met the assumptions of the tests used unless otherwise noted. Data are reported as means ± SEM with α set at 0.05. Hatching success, time to LOE under hypoxia, and condition factor were analyzed as dependent variables using a two-way analysis of variance (ANOVA) with temperature and oxygen saturation as fixed factors. Post hoc tests for two-way ANOVA were carried out using TukeyHSD. CTMax was analyzed using a three-way ANOVA with temperature, oxygen saturation, and developmental stage as fixed factors. Since different CTMax methodologies were used for embryo stages (cardiac cessation [eyed and silver-eyed]) and post-hatch stages (LOE [alevin and fry]), a separate ANOVA was conducted for each. A type III ANOVA was used for the post-hatch stages to account for unequal sample sizes and interactions between the main factors. Post hoc tests for three-way ANOVA were carried out using a Tukey’s test (‘lsmeans’ package, Lenth, 2016). Initial models nested fish within their corresponding replicate treatment buckets; however, with no significant effects, replicate was removed as a factor to reduce models to their simplest form. Condition factor of alevins did not meet assumptions of homogeneity of variance and was log transformed.

## Results

### Hatching success

**Figure 1:**
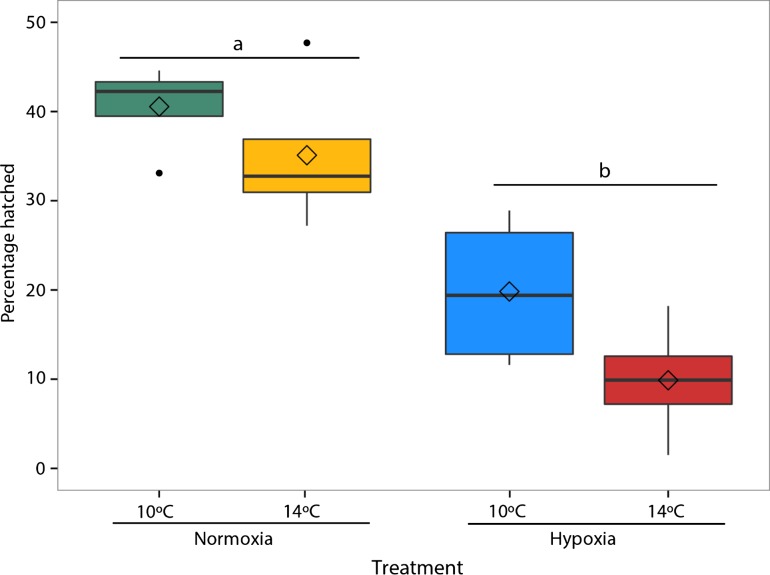
Hatching success measured as percentage hatched in each treatment (10°C Normoxia [green], 10°C Hypoxia [blue], 14°C Normoxia [yellow], and 14°C Hypoxia [red]). The center line of the boxplots represents the median, the box represents the inter-quartile range (IQR), the whiskers extend 1.5 times IQR, black points represent values outside 1.5 the IQR, and diamonds represent the mean. Letters indicate a significant (*P* < 0.05) difference between dissolved oxygen treatments.

Rearing under hypoxia significantly reduced the percentage hatched (*F*_1,12_ = 37.3, *P* < 0.001). Percentage hatched was highest for embryos reared in normoxia with ~40% (40.5 ± 2.6) hatching success at 10°C and ~35% (35.1 ± 4.4) hatching success at 14°C (Fig. 1). At 10°C, embryos reared in the hypoxia treatment had 50% lower hatching success compared to the normoxia treatment (19.8% ± 4.4 vs. 40.5%). Although temperature did not significantly affect hatching success (*F*_1,12_ = 4.19, *P* = 0.06) and there was no significant interaction between temperature and oxygen (*F*_1,12_ = 0.36, *P* = 0.56), hatching success was lowest in the multiple stressor treatment of hypoxia and 14°C with only ~10% hatched (9.9% ± 3.4).

### Upper thermal tolerance

#### Embryos

**Figure 2:**
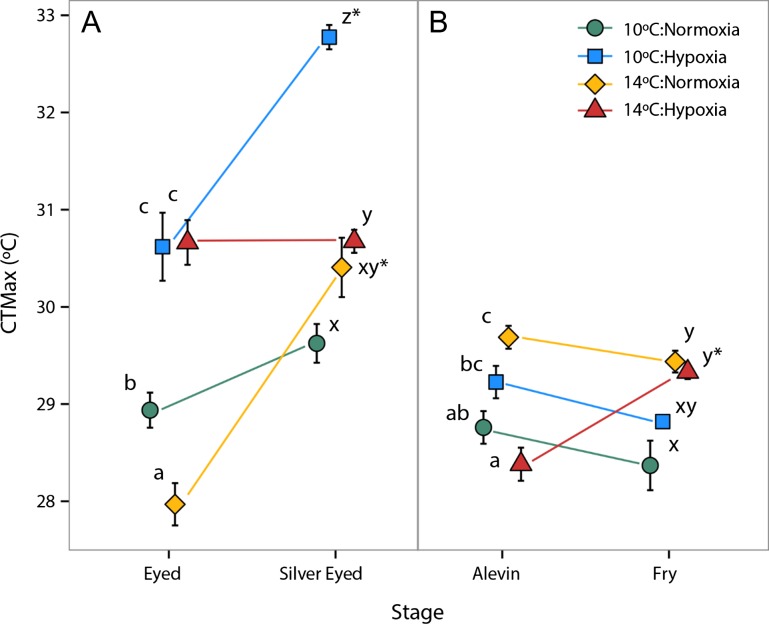
Critical thermal maximum (CTMax) throughout development in four rearing treatments: 10°C Normoxia (green circle), 10°C Hypoxia (blue square), 14°C Normoxia (yellow) and 14°C Hypoxia (red diamond). Average CTMax ± S.E.M. is given for *n* = 16 individuals per treatment (*n* = 15 10°C normoxia alevins, *n* = 8 14°C normoxia fry) at each developmental stage. Within each panel CTMax is defined as (**A**) the temperature at which the heart beat stopped (embryonic stages, eyed and silver eyed) and (**B**) the temperature at which equilibrium was lost (larval stages, alevin and fry). Letters indicate significant (*P* < 0.05) differences between treatments within a given developmental stage. Asterisks indicate significant (*P* < 0.05) differences between developmental stages within a single treatment.

Upper thermal tolerance was highly variable across treatments and development (Fig. 2A). There was a significant two-way interaction between temperature and oxygen (*F*_1, 120_ = 8.36, *P* = 0.005). In addition, a significant three-way interaction (*F*_1,120_ = 36.30, *P* < 0.001) between the main effects of temperature (*F*_1, 120_ = 12.05, *P* < 0.001), oxygen saturation (*F*_1, 120_ = 145.44, *P* < 0.001) and developmental stage (*F*_1, 120_ = 67.1, *P* < 0.001) indicated salmon CTMax was dependent on the life stage and stressors. For example, eyed stage embryos reared under hypoxia at both temperatures had the highest thermal tolerance with a CTMax of 30.6°C ± 0.6 at 10°C and 30.7°C ± 0.2 at 14°C. Eyed embryos reared at 14°C in normoxia had the lowest CTMax (27.9°C ± 0.2) and 10°C normoxia reared embryos had an intermediate thermal tolerance (28.9°C ± 0.2). Thermal tolerance significantly increased at the silver eyed stage for 10°C hypoxia and 14°C normoxia treatments. The 10°C hypoxia treatment had the highest CTMax (32.8°C ± 0.1) with both hypoxia treatments again being the most thermally tolerant. Silver eyed embryos in the 10°C normoxia treatment had the lowest CTMax (29.6°C ± 0.2) and 14°C normoxia was intermediate (30.4°C ± 0.3).

#### Alevins and fry

There were significant interactions between temperature, oxygen saturation, and developmental stage (*F*_1,111_ = 7.68, *P* = 0.007) in the thermal tolerance of the post-hatch alevin and fry stages (Fig. 2B). There were significant two-way interactions between temperature and oxygen (*F*_1,111_ = 35.03, *P* < 0.001) and temperature and stage (*F*_1,111_ = 21.5, *P* < 0.001. There were also significant main effects of temperature (*F*_1, 111_ = 16.9, *P* < 0.001), oxygen (*F*_1, 111_ = 5.32, *P* = 0.02), and stage (*F*_1, 111_ = 4.53, *P* = 0.04) on CTMax. At the alevin stage, the normoxia and hypoxia treatments at 14°C had the highest (29.7°C ± 0.1) and lowest CTMax (28.4°C ± 0.2), respectively, with the alevins reared at 10°C having intermediate CTMax (29.2°C ± 0.2 vs. 28.8°C ± 0.2 for hypoxia and normoxia treatments, respectively). Upon reaching the fry stage CTMax significantly increased in only the 14°C hypoxia treatment (increased to 29.3°C ± 0.1) such that it was no longer significantly different from the 14°C normoxia treatment. Both 14°C treatments had the highest CTMax, while the 10°C normoxia treatment had the lowest (28.4°C ± 0.3) CTMax.

#### Fry hypoxia tolerance

**Figure 3:**
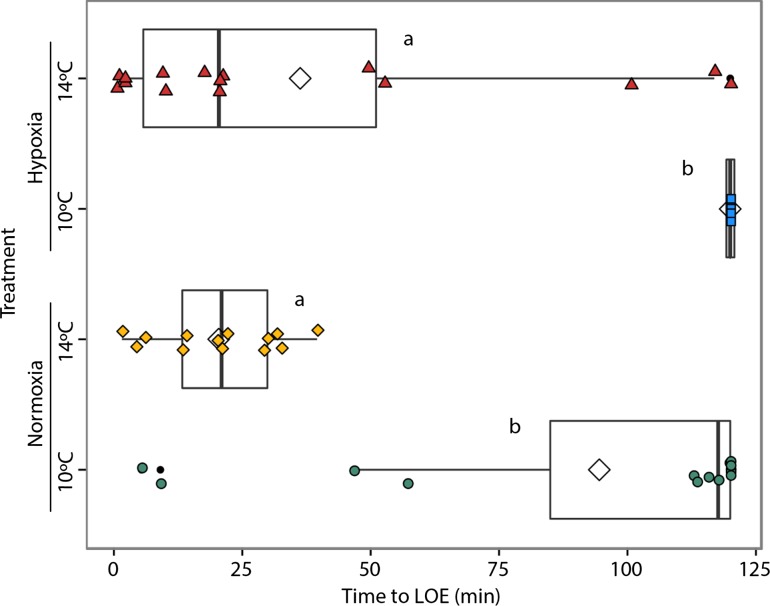
Acute hypoxia tolerance of fry was measured as the time (min) until fish lost equilibrium while held at 8% dissolved oxygen saturation. A total of *n* = 16 individuals per treatment (10°C Normoxia [green, *n* = 15], 10°C Hypoxia [blue *n* = 16], 14°C Normoxia [yellow, *n* = 13] and 14°C Hypoxia [red, *n* = 13]) were tested. Each test was conducted at the temperature fish were reared at and began at the dissolved oxygen saturation of the corresponding treatment. The center line of the boxplots represents the median, the box represents the inter-quartile range (IQR), the whiskers extend 1.5 times IQR, black points represent values outside 1.5 the IQR, and diamonds represent the mean. Letters indicate significant (*P* < 0.05) differences between treatments.

Hypoxia tolerance was only measured at the fry stage, when the fish had absorbed nearly all of the yolk sac. Rearing in hypoxia significantly increased time to LOE (*F*_1,54_ = 6.49, *P* = 0.014) while rearing at 14°C significantly decreased time to LOE (*F*_1,54_ = 91.74, *P* < 0.001) (Fig. 3). Oxygen and temperature did not significantly interact (*F*_1,54_ = 0.35, *P* = 0.56). Fish reared at 14°C in normoxia maintained equilibrium for ~20 min (20.4 ± 3.3) compared to ~36 min (36.3 ± 12) for fry reared at 14°C in hypoxia and ~94.5 min (±11) for fry reared at 10°C in normoxia. Fry reared at 10°C in hypoxia were the most tolerant to hypoxia and all maintained equilibrium indefinitely during the 2-h trial period at 8% air saturation (120 min).

### Growth

**Figure 4:**
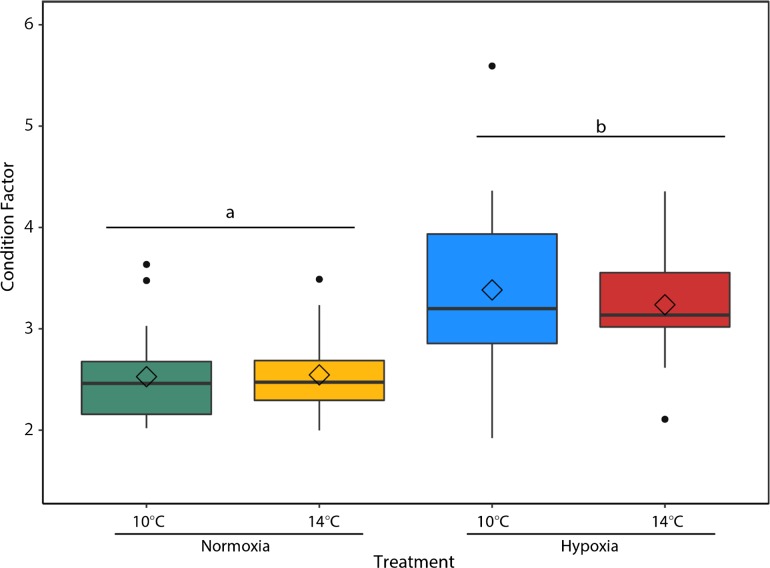
Fulton’s condition factor in post-hatch alevins was calculated as the relationship between mass and length in *n* = 20 individuals per treatment (10°C Normoxia [green], 10°C Hypoxia [blue], 14°C Normoxia [yellow] and 14°C Hypoxia [red]). The center line of the boxplots represents the median, the box represents the inter-quartile range (IQR), the whiskers extend 1.5 times IQR, black points represent values outside 1.5 the IQR, and diamonds represent the mean. Letters indicate a significant (*P* < 0.05) difference between the main effects of dissolved oxygen (Normoxia, and Hypoxia).

**Figure 5:**
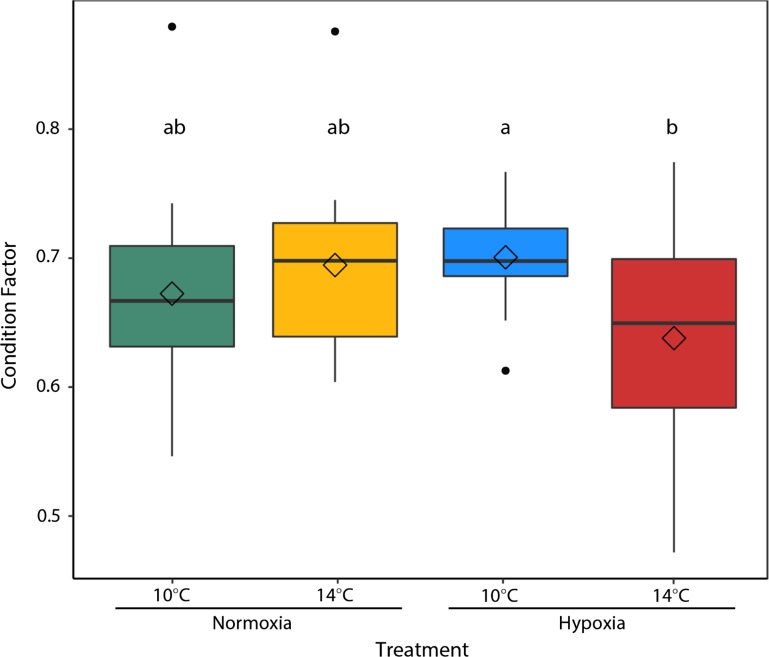
Fulton’s condition factor in fry was calculated as the relationship between mass and length in *n* = 20 individuals per treatment. The center line of the boxplots represents the median, the box represents the inter-quartile range (IQR), the whiskers extend 1.5 times IQR, black points represent values outside 1.5 the IQR, and diamonds represent the mean. Letters indicate significant differences between treatments (10°C Normoxia [green], 10°C Hypoxia [blue], 14°C Normoxia [yellow], and 14°C Hypoxia [red]).

Alevins reared in hypoxia had a significantly higher Fulton’s condition factor (*F*_1,75_ = 37.51, *P* < 0.001) compared to alevins reared under normoxic conditions (Fig. 4). There was no significant interaction between temperature and oxygen on alevin condition factor (*F*_1,75_ = 0.39, *P* = 0.53). Upon reaching the fry stage there were no significant differences in condition factor between treatments. Temperature (*F*_1,70_ = 1.004, *P* = 0.32) and oxygen saturation (*F*_1,70_ = 0.44, *P* = 0.51) did not significantly affect Fulton’s condition factor, although there was a significant interaction between the two stressors (*F*_1,70_ = 7.62, *P* = 0.007) (Fig. 5), where warming decreased condition factor in hypoxia-reared fish.

### Developmental rate

Developmental rate was assessed at the treatment level because there was very little variation between replicate buckets within a treatment. Fish developed faster at 14°C (Table 2). Under normoxia, fish reared at 14°C reached each stage 7–10 days before fish reared at 10°C. Rearing in hypoxia further delayed development within each temperature. At 14°C rearing in hypoxia delayed development by 4–6 days depending on the stage, although hypoxia-reared fish hatched just one day after normoxia-reared fish. At 10°C fish reared in hypoxia reached each stage 4–10 days later than in normoxia, depending on the stage.

**Table 2:** Time (days post fertilization) for 50% of individuals or more in each treatment to reach four developmental stages. Development was assessed daily in all replicate culture buckets per treatment.

	Time (days post fertilization) to reach stage			
Treatment	Eyed	Silver Eyed	Post-hatch	Fry
14°C Normoxia	17	26	35	64
14°C Hypoxia	21	30	36	70
10°C Normoxia	25	36	42	75
10°C Hypoxia	29	41	48	85

## Discussion

This study investigated how Chinook salmon development is influenced by the interaction between warming and hypoxia. Acclimation to elevated temperature and hypoxia improved acute thermal tolerance and hypoxia acclimation also improved tolerance to acute hypoxic stress, suggesting a capacity to acclimate to warming and hypoxia during early life stages. Despite improved physiological tolerance with chronic rearing under elevated temperature and hypoxia, hypoxia reduced early growth and hatching success, especially in combination with warming. Reduced growth and hatching success could lead to detrimental effects at the population level as climate change progresses.

### Hatching success

The hatching process in fish embryos is a critical period during development and is strongly influenced by both temperature and oxygen (Yamagami, 1988; Korwin-Kossakowski, 2012). In the present study, warm temperature alone minimally reduced hatching compared to controls, which is not surprising given that California Central Valley Chinook salmon embryos were found to tolerate temperatures up to 16°C in laboratory studies (Myrick and Cech, 2004; Williams, 2006). Fish embryos are particularly susceptible to low DO in their environment during the critical period of hatching (Keckeis *et al.*, 1996). Here, rearing in hypoxia markedly reduced hatching success at both temperatures (Fig. 1), with the majority of this mortality occurring within a day or two of the mean hatch date for a given treatment. The observed mortality at hatch is consistent with observations in hypoxia-reared lake trout (Garside, 1959; Carlson and Siefert, 1974) and largemouth bass (Dudley and Eipper, 1975). The mortality observed at hatch often occurred in partially hatched embryos where individuals were able to free their heads from the chorion but were unable to fully escape, suggesting the physical process of hatching was more challenging in hypoxia.

Hatching is an energetically costly process due to increased movement and oxygen consumption (Hamor and Garside, 1959; Ninness *et al.*, 2006). With a limited capacity for anaerobic metabolism in embryos (Rombough, 2011), hatching may increase aerobic energy demand to a level that cannot be matched by energy supply under hypoxic conditions (Polymeropoulos *et al.*, 2016). Warmer water temperatures increase the metabolic rate, and thus oxygen demand, of embryos. Combined with the additional energy required for hatching, the mismatch between energy supply and demand may have been greatest in the multiple stressor treatment of 14°C hypoxia (Pan and von Herbing, 2017), which had the lowest hatching success (Fig. 1). Low hatching success due to rearing in hypoxia and warming may be problematic for salmon populations as small changes in the survival of early life stages can have large effects on recruitment and adult population size (Trippel and Chambers, 1997). Of note, embryos reared in normoxia at 10°C had unexpectedly low hatching success for control conditions (~40%). The low percentage hatched in the control treatment was likely influenced by unusually high mortality observed in one family of embryos, possibly due to poor embryo quality.

### Upper thermal tolerance

Many fish species have some degree of plasticity in thermal tolerance (Beitinger *et al.*, 2000), such that upper thermal tolerance commonly increases with acclimation to warmer temperatures (e.g. Fangue *et al.* 2006; Healy and Schulte, 2012; Anttila *et al.*, 2015). Consistent with results from other studies of warm acclimation in fishes, alevins and fry reared at 14°C under normoxia had the highest CTMax. In contrast to what would be predicted, eyed embryos (the first stage measured) reared at 14°C in normoxia had the lowest CTMax (Fig. 2). Given the high mortality of 14°C normoxia-reared fry after CTMax trials, fish reared at 14°C may have been near their thermal limit such that they were less able to allocate energy to stress tolerance mechanisms to the extent that 10°C treatments could. Thermal tolerance is often life stage specific (Komoroske *et al.*, 2014), particularly in fishes that occupy different habitats throughout development such as Pacific salmon (McCullough, 1999; Richter and Kolmes, 2005). Salmon embryos develop in cold streams and are therefore likely to be more sensitive to warming at this stage.

Oxygen limitation of thermal tolerance hypothesizes that CTMax will be lower when exposed to environmental hypoxia. In contrast, the CTMax of 10°C hypoxia-reared embryos and alevins in the present study were consistently higher than the CTMax of 10°C normoxia-reared fish at all developmental stages. Alevins and fry reared at 10°C in hypoxia maintained a higher CTMax compared to 10°C normoxia reared fish, but had a lower CTMax than fry reared at 14°C in either oxygen treatment suggesting a stronger effect of acclimation temperature on the thermal tolerance of post-hatch stages. Although CTMax often decreases in hypoxia (e.g. Rutledge and Beitinger, 1989; Healy and Schulte, 2012; Ellis *et al.*, 2013), CTMax has also been shown to be independent of oxygen availability (e.g. Ern *et al.*, 2016; Motyka *et al.*, 2017; Verberk *et al.*, 2018). CTMax can be maintained in moderate levels of hypoxia, such as those maintained in this study, even in stenothermal species (Ern *et al.*, 2017); however, the improvement of CTMax with acclimation to hypoxia as observed in the present study is unexpected.

The multiple stressor treatment of 14°C hypoxia had a relatively high CTMax throughout development with the exception of the alevin stage, which had the lowest CTMax for that stage. Mechanisms to cope with hypoxia include adjustments to increase oxygen uptake at the gills and improve transport to increase the supply of oxygen to tissues, as well as reductions in metabolic rate to decrease oxygen demand (Miller *et al.*, 2008; Richards, 2009; Polymeropoulos *et al.*, 2016). Since upper thermal tolerance can benefit from improved oxygen delivery, the mechanisms underlying long-term acclimation to hypoxia can also maintain or improve thermal tolerance (Burleson and Silva, 2011; Motyka *et al.*, 2017) and the physiological adjustments made could have been responsible for increased upper thermal tolerance seen in this study. It should be noted that all CTMax trials were conducted in normoxic conditions, so embryos acclimated to hypoxia may have been more thermally tolerant in part because of an increased availability of oxygen during the CTMax trials, compared to acclimation conditions.

### Hypoxia tolerance in fry

Within the OCLTT framework elevated temperatures are predicted to decrease tolerance to acute hypoxia (McBryan *et al.*, 2013). Consistent with the OCLTT, the time to loss of equilibrium in hypoxia was significantly shorter in fish reared at 14°C compared to 10°C, indicating reduced hypoxia tolerance with warming (Fig. 3). Lower hypoxia tolerance at warmer temperatures has been observed in many other studies (Nilsson *et al.*, 2010; Barnes *et al.*, 2011; Remen *et al.*, 2013; McDonnell and Chapman, 2015; Borowiec *et al.*, 2016), although it varies by species (e.g. He *et al.*, 2015). Higher temperatures are thought to reduce hypoxia tolerance by increasing metabolic rates and in turn, oxygen demand (Pörtner, 2010), and may also decrease the oxygen binding affinity of hemoglobin, thereby reducing oxygen supply (McBryan *et al.*, 2013).

Rearing in hypoxia improved tolerance to acute hypoxia at both temperatures compared to the normoxia treatments. Improvement of hypoxia tolerance following acute (24–48 h) exposure and longer-term acclimation occurs in many fishes (e.g. Rees *et al.*, 2001; Timmerman and Chapman, 2004; Shimps *et al.*, 2005; Fu *et al.*, 2011). Acclimation to hypoxia can involve a number of mechanisms such as improved oxygen uptake and transport through changes in gill morphology, concentration of red blood cells and hemoglobin, as well as alterations to cellular energy metabolism (Farrell and Richards, 2009; Borowiec *et al.* 2015). Additionally, there was high individual variability in hypoxia tolerance in the 14°C hypoxia and 10°C normoxia treatments. This variability may be due to the contrasting effects of warming and hypoxia on hypoxia tolerance such that individual responses to exposure to one factor that improves tolerance (i.e. low temperature or hypoxia acclimation) in combination with a factor that reduces hypoxia tolerance (i.e. elevated temperature or normoxia acclimation) are more variable than exposure to two factors that both either increase or decrease tolerance. Our results suggest that Chinook salmon fry also have the capacity to acclimate to hypoxia during chronic exposure, although the degree of improved hypoxia tolerance is temperature dependent.

### Growth and development

Reduced growth and delayed development in hypoxia are compensatory responses where metabolic demand is adjusted to match the oxygen supply available (Rombough, 1988a). Despite having higher condition factors, hypoxia-reared alevins were smaller due to reduced body tissue length (data not shown) and more yolk retained at the time of hatch (Fig. 4), similar to Polymeropoulos *et al.* (2017) with hypoxia-reared Atlantic salmon. A reduction in size of post-hatch hypoxia-reared larvae has been observed in many other studies (Alderdice *et al.*, 1958; Garside, 1959; Shumway *et al.*, 1964; Marks *et al.* 2012). Growth is the most energetically demanding activity in early embryonic development and is almost entirely dependent on aerobic metabolism (Rombough, 2011). While the ecological significance of size at hatch is difficult to determine, alevins that are smaller at hatch may have lower chances of survival due to size selective predation pressure, decreased competitive ability, and slower swimming speeds (Mason, 1969; Pepin, 1991). Smaller salmon may be more vulnerable to predation in the Sacramento-San Joaquin Delta where predation on juvenile Chinook salmon by abundant native and non-native fish predators is high (Grossman, 2016). Given the challenges of predicting the effects of size on survival, size is best considered alongside performance (Conover and Schultz, 1997; Green and Fisher, 2004). For fish that do survive hatching in hypoxia there is a potential tradeoff between a smaller size at hatch and being more tolerant to acute thermal and hypoxic stressors. Upon reaching the fry stage there were no significant differences in condition factor between treatments (Fig. 5); however, it took hypoxia reared fry 6–10 days longer to reach the fry stage and fully absorb the yolk sac.

Developmental rate in fish embryos is highly dependent on both temperature and DO in the rearing environment (Murray and McPhail, 1987; Beacham and Murray, 1990). Both decreased temperature and hypoxia lead to slower development in many fish species (Garside, 1966; Pepin, 1991; Green and Fisher, 2004). As expected, in the present study low temperature delayed development by 7–10 days in 10°C normoxia compared to 14°C normoxia (Table 2). The developmental delay in hypoxia increased from 4 to 5 days during the embryonic stages to 6–10 days to reach the post-hatch stages, as in Geist *et al.* (2006), with the exception of the 14°C hypoxia treatment. The further delay of developmental rate in hypoxia may have larger phenological consequences as there may be selection against late emerging salmon (Einum and Fleming, 2000).

Low oxygen can have two opposite effects on time to hatch (Carlson and Siefert, 1974; Ciuhandu *et al.*, 2005; Hassell *et al.*, 2008), both of which appear to have occurred in this study, dependent on rearing temperature. Hypoxia can slow the overall rate of development extending the time to hatch (i.e. 10°C hypoxia treatment hatched 6 days after the 10°C normoxia treatment). Hypoxia can also reduce the time to hatch. Low oxygen is an important natural signal to hatch in fish embryos (Czerkies *et al.*, 2001) and acute hypoxia can trigger hatching in mature embryos (Oppen-Berntsen *et al.*, 1990). As embryonic development progresses, metabolic rate increases until ambient oxygen can no longer meet metabolic oxygen demand (Rombough, 1988b). Thus, hypoxia can trigger premature hatching when oxygen becomes limited before embryos are fully developed (DiMichele and Powers, 1984; Latham and Just, 1989). Given the increase in metabolic demand with warming, early oxygen limitation may explain why embryos reared at 14°C in hypoxia hatched just one day after those in 14°C normoxia, when the hypoxia treatment reached all other stages multiple days later. Similarly, precocious hatching resulting from acute hypoxia exposure was greatest at high temperature in whitefish embryos (Czerkies *et al.*, 2001).

## Conclusions

Late fall-run Chinook salmon in the Central Valley of California are listed as a Species of Concern under the federal Endangered Species Act and occupy some of the same river habitat as threatened and endangered Chinook salmon runs (i.e. threatened spring-run and endangered winter-run). A modeling study by Martin *et al.* (2017) suggested interactions between high temperatures, low flows, and low DO may have contributed to high embryo mortality in winter-run Chinook salmon, a run with a population of less than 1 000 estimated to be in the Sacramento River during the 2017 spawning season (Azat, 2018). Survival of wild Central Valley salmon embryos can be highly variable but is generally low, with average egg to fry survival likely below 20% (Williams, 2006). A further decrease in hatching success resulting from hypoxia, as demonstrated in this study, could potentially have large impacts on population size as a whole if hypoxia is widespread throughout the rearing habitat. Furthermore, hypoxia and warming affected many aspects of salmon development as both individual and interacting stressors in this study, suggesting both factors should be considered in the conservation and management of early life stage Chinook salmon. Exposure to warming or hypoxia during early development of fishes were found to influence long-term behavior (Ivy *et al.*, 2016; Roussel, 2007), swimming performance (Widmer *et al.*, 2006; Johnston *et al.*, 2013), stress tolerance (Zambonino-Infante *et al.*, 2013), growth and physiology (Crocker *et al.*, 2013; Zambonino-Infante *et al.*, 2017; Cadiz *et al.*, 2018) and adult salmon migration timing (Jonsson and Jonsson, 2018). While current management strategies to promote embryo survival in the Sacramento River are largely focused on releases of cold water from the Shasta Dam to maintain temperatures at or below a target temperature of 56°F (~13.3°C) (National Marine Fisheries Service, 2009); this study demonstrates the need to also manage dissolved oxygen (in addition to temperature) when regulating flows as both factors influence the survival and development of salmon embryos. This study, in addition to Martin *et al.* (2017), suggests that in natural redds where DO is variable, the target temperature of 56°F may be too high in some cases since salmon embryo mortality can occur at lower temperatures in hypoxia. The exact mechanisms underlying the acclimation capacity at these early stages, as well as the potential for persistent or latent physiological effects of exposure to warming and hypoxia during early development warrant further investigation.
